# Under What Conditions Can the Nasal Douche Be Employed without Danger*A paper read before the Medico-Chirurgical Society of Berlin, Dec. 28, 1877.

**Published:** 1878-09

**Authors:** 

**Affiliations:** Lecturer on Otology at the University of Berlin


### Translation.

### UNDER WHAT CONDITIONS CAN THE NASAL DOUCHE BE EMPLOYED WITHOUT DANGER?*

By Dr. Weber-Liel.

*(Lecturer on Otology at the University of Berlin.)*

(*Translated at the Author's request by* F. C. Hotz.)

Gentlemen: You are aware that the first requirement in the treatment of affections of the naso-pharyngeal space is the frequent and gentle cleansing of the diseased mucous membrane. It is not only desirable, but almost always necessary that the medicaments be applied to parts which we cannot directly reach with instruments.

Theodore Weber's nasal douche seemed, in many respects, to accomplish this purpose; and aural surgeons everywhere employed it. The favorable results obtained with it, accounted for its growing popularity. Recently, however, many physicians have changed their opinion of the usefulness of this hitherto popular instrument, as a great number of cases were published, in which the use of the nasal douche was followed by violent aural inflammations, sometimes jeopardizing the life of the patient. These observations lead to the conclusion that the inflammation was caused by the entrance of some of the fluid which passed through the nose, into the Eustachian tube and tympanic cavity. Some, considering this accident unavoidable, discountenance the use of the nasal douche altogether; while other surgeons believe they

* A paper read before the Medico-Chirurgical Society of Berlin, Dec. 28, 1877.

can obviate the entrance of fluid into the middle ear by certain precautions and by modifications of the douche.

The fact is that all American, English and German surgeons who have written on this subject, only consider the possibility or impossibility of preventing fluid from entering the middle ear. *They entirely forget to ventilate the question whether the entrance of fluid into the middle ear always causes an inflammation.* I shall presently show that this is not the case, but that the occurrence or non-occurrence of otitis after the entrance of fluid into the middle ear is determined :

(*a.*) By the quality of the injected fluid.

(*b.*) By the state of the mucous membrane of the naso-pharyngeal space, Eustachian tube and tympanic cavity at the time when the douche is applied.

(*c.*) By the behavior of the patient during and directly after the douche.

But let me first say a few words in regard to the various methods of douching the naso-pharyngeal space.

I presume you all are familiar with Th. Weber's nasal douche and its application. It will, therefore, be sufficient for our purpose to draw your attention to the fact that its current of water is not uniform and continuous ; it is gradually growing weaker, because the column of water in the vessel is steadily decreasing, and it is often interrupted, because the hand holding the tube cannot be kept absolutely steady and immovable. For these reasons, we notice during the operation of the douche the contraction of the visible musculature of the palate is not uniform, but the velum and the posterior arches are in a trembling motion of constantly changing tension. But if it is the levator veli muscle which bars the introitus tubæ against the entrance of fluid as it flows by, we can conceive the possibility of its entering the tuba at the moments of decreased muscular tension. I have also shown, by experiments with the different kinds of nasal douches, that they cannot be used longer than half a minute without sneezing or swallowing. By these acts the mouth of the tuba is opened, and the fluid enters it, unless the douche be instantaneously discontinued. Still greater is the possibility that fluid will get into the middle ear if a Davison's syringe is employed for the douche. The fluid is thrown into the nostrils by jets and the flow is very irregular and variable in force, and consequently the state of contraction of the muscles varies constantly. The danger is decidedly lessened by following Dr. Fraenkel's advice, which is, that one should begin to sound the German vowel u (oo in English) before the water is started, and continue the pronunciation of this vowel as long as the water is passing through the nostrils. During this phonation of u, the levator palati is very strongly contracted, and its belly is crowded against and into the ostium pharyngeum tubæ. But if the closure of the isthmus of the pharynx is overcome, as it might be at any time when the pressure of the water is suddenly increased by a stronger compression of the rubber bulb; if then deglutition becomes a necessity, it would require a great deal of practice and presence of mind of a patient, to stop the douche before he stopped sounding.

For many years I have been using the syringe for nasal douche. My naso-pharyngeal syringe is of glass, so that I can see whether its contents are free from impurities; it holds just enough water to fill the naso-pharyngeal cavity of an adult. Its bulbous nozzle is of hard rubber; the piston is of metal, and the piston rod ends in a ring for the thumb. The injections are made in the following manner: the head of the patient being held up as straight as possible and steadied by the physician's one hand, the nozzle of the syringe is inserted into that nostril which on a previous examination was found the less pervious; the fluid will thus find no difficulty in flowing off through the other nostril. If we watch the arches and velum of the palate during an injection into the nostril, we shall observe the posterior arches by a sudden contraction, approach each other, and the posterior wall of the pharynx; we shall further observe how, by an energetic reflex contraction of its musculature, the velum is drawn upwards, and its posterior edge laid firmly against the posterior pharyngeal wall. We shall not observe any tremulous movements or unequal variable tension of the parts, as a careful inspection detects when the other modes of the douche are employed. This powerful contraction of the levator palati beginning and ending with the injection, so thoroughly occludes the mouth of the Eustachian tube, that no fluid can be forced into it. If the fluid is ejected from the syringe with less pressure, the reactionary contraction of the muscle is less strong accordingly, but still sufficient to keep the fluid out of the Eustachian tube. There is another advantage the injection with the syringe has over the nasal douche. With the syringe we are able to reach and cleanse thoroughly all parts of the naso-pharyngeal space, while experiments on dead bodies have convinced me that the fluid thrown into the nostrils with Weber's douche flows over the parts in the immediate vicinity of the posterior nares, but never reaches the roof of the naso-pharyngeal cavity. It is, however, not always allowed to execute the douche, in the described manner. In certain persons the fluid, though thrown into the nostrils with moderate force, enters the middle ear comparatively easily. We shall find in such cases that during the act of deglutition or phonation the soft palate is incompletely raised. During the douche, then, such persons can scarcely abstain from swallowing, because the water runs down behind the velum instead of returning through the other nostril. The rhinoscopic examination of some such persons has shown me that where the naso-pharyngeal space was insufficiently closed against the fauces, the ostium tubæ was imperfectly occluded by the contracted levator muscle. Children have a very short and wide Eustachian tube; and the fluid when injected even with very little force, often enters the middle ear, and therefore we have to be particularly cautious; although the experiment shows that in children the entrance of fluid into the middle ear is seldom followed by inflammation. On the other hand, there are numerous cases where the most forcible injection, or any other mode of douching, applied most improperly and carelessly, does not cause the entrance of fluid into the tuba. They are those cases in which the wall of the Eustachian tube is collapsed from inefficiency of its musculature, or its lumen narrowed by catarrhal swelling of its mucous membrane. This point can be ascertained by means of bougies and the air-bath, and is of great importance in deciding the question whether and how in a given case the nasal douche can be employed.

Speaking now of the conditions under which an inflammation supervenes upon the entrance of fluid into the middle ear, I have first to state that all publications on this subject have overlooked one, and in my opinion the most essential point.

We must ask the question : Do *all* fluids exert so noxious an influence upon the middle ear that their entrance must result in a violent inflammation ?

Gruber stated that after injections of medicated fluids into the middle ear, he often observed symptoms of irritation rather than inflammation. And further I beg to mention that in the "*Deutsche Klinik*," No. 3, 1866, and "*Monatsschrift für Ohrenheilkunde*," Nos. 4 and 6, 1870. I communicated the fact that different fluids caused different degrees of inflammation ; that, for instance, injections of sulphate of copper or zinc were followed by violent otitis media, but never caused perforation of the membrana tympani, while silver nitrate or corrosive sublimate caused a violent otitis with rapid perforation of the drum head.

During the past year Dr. Acker, of Washington, D. C., and I made a series of experiments on animals to test the effect different medicines would have upon the middle ear if they get there by the way of the Eustachian tube.

The result of these experiments (which Dr. Acker described in his dissertation Ueber Injectionen in den Nasenrachenraum und in die Tube, Berlin, 1877,) was as follows : "If we injected with some force a colored fluid into one nostril of a rabbit, the other nostril being closed, we could always find, at the post mortem some of the fluid in the middle ear, but if the other nostril was *not* closed during the injection, the fluid returned in a stream through the nose, and the autopsy confirmed that no fluid had entered the tympanic cavity. Numerous experiments with warm solutions of salt in various dilutions showed that shortly after the entrance of salt water into the middle ear a violent inflammation was developed. The vessels of the promontory and membrana tympani were highly distended ; the secretion was increased and contained large numbers of white blood cells. After some time perforation of the membrana tympani took place. In all the animals thus experimented upon, the membrana tympani was perforated, and in one rabbit the tympanic cavities and both external meatus were filled with a fibrinopurulent exudation. Injections of weak solutions of nitrate of silver were also followed by purulent otitis media with perforation of the membrana tympani.

But the results were entirely different, if we used solutions of soda carbonate for injections. In no case it resulted in the perforation of the membrana tympani, although in some tympana the traces of a past inflammation could be positively discovered.

These results fully agree with the observations made in men. And I would draw your attention particularly to the fact *that salt water had been employed in all cases where dangerous otitis media supervened.* The use of the nasal douche in men, just as the injections of salt water in animals, caused otitis media acuta with perforation. On the other hand, the experiments with pure solutions of soda prove that this is a far less dangerous remedy for the middle ear. It is only through a combination of other noxious influences that strong solutions of soda may perhaps cause subacute inflammation in the tympanum ; but I nave never observed such accidents after soda injections. By means of my tympanic catheter I am able to force the solution directly into the tympanic cavity. I have practiced this mode of treatment a number of years for different affections of the middle ear, and I can positively affirm that the introduction of soda solution into the tympanic cavity has not in a single case been followed by pain or inflammation. One might say it is uncertain whether, in this treatment, the medicine gets into the middle ear at all. This objection is easily met by the following experiment: The medicine being colored by a small addition of aniline red (which must be free from arsenic) the tympanic catheter is introduced through the silver Eustachian catheter into the tympanic cavity, and the fluid forced into the tympanic cavity by strong compressions of the air bag. After this proceedure the presence of the colored fluid behind the membrana tympani can easily be confirmed by the aniline color, which is especially marked at the anterior portion of the drumhead.*

* For the observation that a fluid colored with pure aniline can be injected in the above manner without fear and danger, I am indebted to one of my students, who, while practicing the use of the tympanic catheter one day, by mistake, filled it with a weak solution of aniline. The case caused me a great deal of anxiety, but it passed off without any reaction.

During the years that I have been using warm soda lotions for the nasal douche, I have not observed any unpleasant symptoms of inflammation after the douche, if the patients conducted themselves according to prescribed rules.

My suggestion, therefore, is to abandon the salt water and to try a weak solution of soda instead.*

I have no doubt but that the occurrence of otitis media after the use of the nasal douche depends as much on the state of the mucous membrane of the naso-pharyngeal space and the Eustachian tube as on the state of the tympanic cavity. Almost all aural surgeons will agree with me that injections into the naso-pharyngeal space may be made without danger in case of suppurative otitis with perforation of the membrana tympani. The fluid injected through the nostril passes through the aperture in the membrana tympani, and escapes through the external meatus; even when salt water had been used, I have never observed a reactive otitis media under such conditions, provided the patient had guarded himself against taking cold by plugging the external meatus.

On the other hand, the nasal douche becomes a serious matter if the mucous membranes are in a state of increased irritability or sub-acute inflammation, as, for instance, if a naso-pharyngeal catarrh is just developing. At such times the irritation caused by the nasal douche is certain to aggravate the inflammatory state of the mucous membrane, and the slightest cold will incite a violent inflammation of the middle ear; because the mucous membrane of the Eustachian tube and tympanic cavity readily participates in the inflammatory actions of the naso-pharyngeal space.

Once, while suffering from a cold in my head, I demonstrated on my own person, the use of the nasal douche. I am certain that no fluid entered the tympanic cavity—a fact easily decided by an expert, who, from daily experiments, is familiar with the sensations in the ear caused by the entrance of fluids, introduction of a bougie, etc.

Nor did I experience anything directly after the injection save a burning sensation in the region of the orifice of the right Eustachian

* The solution may be strong enough to give the fluid a decided alkaline taste, about five Grams to 100.

tube. Several hours later deglutition became unpleasant, and I felt an increased warmth and burning sensation in the middle ear. Violent throbbing in the ear supervened and the attempt to blow my nose caused a painful tension of the membrana tympani and the crackling of bubbles of mucous. By keeping the ear warm and plugged, I felt relieved, and all threatening symptoms disappeared, after a day's rest and quiet. But I am convinced, had I neglected these precautionary measures, the irritation would have developed into a violent otitis media, *although the fluid of the nasal douche had not entered the middle ear.*

The symptoms of acute otitis do not follow directly the use of the douche, but appear after an interval of from 4 to 10 hours, after other noxious influences have acted upon the patient. The douche itself simply increases the disposition of the mucous membrane to inflammatory action, so that the supervention of any irritation (as overheating, excitement, cold, etc.) suffices to excite the inflammation. In consideration of this *increased liability* to otistis media the patient should receive strict instruction regarding his behavior after the use of injections into the naso-pharyngeal space. Especially if the patient experiences the slightest feeling of discomfort in the ear immediately after the douche, he must be enjoined to plug and cover the ear, avoid all talking, rest, and above all, to remain at home.*

It has also been mentioned by other writers that the entrance of fluid into the middle ear depends materially on the patient's correct behavior during the use of the douche. He must not use it until he can breathe quietly : he must not speak or swallow, and if the desire for swallowing becomes uncontrolable, he must stop the douche at once. The head must be held perfectly straight, neither bent backwards nor inclined forwards, because in the latter position the injected fluid is likely to get in the frontal sinus. He must not blow his nose directly after the douche, lest in so doing the fluid and mucous which have been gathered at the entrance of the Eustachian tube, be forced on into the tympanic cavity. It is important also, as already mentioned

---

* A careful scrutiny of the published reports on otitis after nasal douche will reveal the fact that some patients were suffering from an attack of coryza at the time, and others exposed themselves carelessly to wind and weather immediately after use of the douche.

by me, to inject the water through the less pervious nostril, so that the return of the water through the other nostril is unimpeded. Fraenkel's suggestion to pronounce the vowel u during the douche is particularly applicable where a wide tuba and an insufficient contraction of the levator palati muscle favor the entrance of fluid into the tube.

But all these points are of less importance compared with the fact, proven by experience and experiments, that the *quality* of the fluid used in nasal douches is the principal agent which determines the occurrence or non-occurrence of otitis media. I am firmly convinced that my colleagues will have no more unpleasant experience with the nasal douche if they use warm solutions of pure carbonate of soda instead of salt water, and at the same time pay more attention to the observation of all the other precautions.

---

We take pleasure in reproducing, upon another page, the excellent letter of Dr. T. K. Cruse to the *N. Y. Medical Record*, (and published in that journal August 10, 1878) upon the subject of medical education in France. Our readers will bear witness to the fact that the editorial and many other pages of the CHICAGO MEDICAL JOURNAL AND EXAMINER have faithfully depicted to the profession the glaring vices of the American system of "educating" medical men, and of appointing them afterward to every manner of official position in colleges, hospitals and the service of cities and States. Dr. Cruse's letter from Paris is such an apt reminder of much that we have said, that we feel glad to publish it in full. In doing this we make an exception to the rule that has been maintained strictly thus far in the conduct of this journal, a rule which has led us to rely upon original work for presentation to our readers.

